# Active Hepatitis C Leading to Refractory Thrombotic Thrombocytopenic Purpura – A Dubious Association and The Challenges Faced in Management.

**DOI:** 10.7759/cureus.5147

**Published:** 2019-07-16

**Authors:** Mounika Gangireddy, Isha Shrimanker, Sandy Saintelia, Vinod K Nookala

**Affiliations:** 1 Internal Medicine, University of Pittsburgh Medical Center - UPMC - Pinnacle, Harrisburg, USA

**Keywords:** refractory thrombotic thrombocytopenic purpura, hepatitis c, rituximab, prednisone, plasma exchange

## Abstract

Acquired thrombotic thrombocytopenic purpura is a combination of thrombocytopenia with microangiopathic hemolytic anemia. A 62-year-old female was transferred from an outside hospital for rapidly worsening mental status and severe thrombocytopenia. Laboratory studies were significant for reduced hemoglobin and platelet count along with raised blood urea nitrogen, creatinine, and serum lactate dehydrogenase levels. Peripheral smear showed numerous schistocytes and further testing showed low ADAMTS13 activity, high ADAMTS13 inhibitor, and positive hepatitis C virus antibody with a high hepatitis C virus ribonucleic acid (RNA) load. The patient was diagnosed with acquired thrombotic thrombocytopenic purpura and started on plasma exchange and steroids. Since no response was achieved until day four of treatment, weekly rituximab was initiated. After the initial two doses of rituximab, she showed significant improvement clinically. ADAMTS13 levels returned back to normal. Cyclosporine was added, following which platelet counts were normalized. Cyclosporine was discontinued, plasma exchange and steroids were slowly tapered off. Follow-up visits showed that the patient is off treatment and continues to be in remission and on regular treatment for hepatitis C. Acquired thrombotic thrombocytopenic purpura is a hematological emergency. Our patient remained refractory to standard therapies and required rituximab and immunosuppressive agents like cyclosporine. We describe the association of active hepatitis C with acquired thrombotic thrombocytopenic purpura that was refractory to plasma exchange, high dose steroids and rituximab. As per our knowledge, this is the first case in the literature to describe a possible association between active hepatitis C and acquired thrombotic thrombocytopenic purpura.

## Introduction

Thrombotic thrombocytopenic purpura (TTP) is defined as thrombotic microangiopathy that is identified by microangiopathic hemolytic anemia (MAHA), consumptive thrombocytopenia, fever, and systemic involvement, especially renal and neurological abnormalities. The classic ‘pentad’ of clinical manifestations described previously is present only in 5% of patients with TTP [1]. In 1924, Eli Moschcowitz first reported the occurrence of TTP in a 16-year-old girl with symptoms of fever, petechiae and neurological involvement along with thrombocytopenia and hemolytic anemia [2].

TTP occurs due to a decrease in the activity of ADAMTS13, which is a von Willebrand factor (vWF)-cleaving protease. Reduced activity of this enzyme results in an aggregation of unusually large vWF multimers. In the presence of shear stress, these uncleaved multimers of vWF result in adhesion of platelets to the damaged endothelial wall of the blood vessel and thus lead to platelet aggregation. This results in the formation of platelet-rich microthrombi that causes occlusion of the vasculature, eventually causing MAHA, thrombocytopenia and visceral ischemia.

The two forms of TTP include hereditary and acquired. The hereditary form is characterized by the presence of mutated genes of ADAMTS13. The acquired form, on the other hand, occurs due to autoantibody formation against ADAMTS13 resulting in reduced activity. Autoimmunity is hypothesized to be secondary to neutralizing antibodies that suppress the proteolytic activity of ADAMTS13 or due to the nonneutralizing antibodies that result in interference with the binding of ADAMTS13 to endothelial surfaces or result in increased ADAMTS13 clearance [3]. 

The 2012 American Society of Apheresis Consensus Conference on TTP described remission when a platelet count >150 000/μL is present for two consecutive days, lactate dehydrogenase (LDH) that is within the normal range and gradual improvement in neurologic symptoms [4]. The mainstay treatment of TTP involves plasma exchange (PLEX) and corticosteroids. 

In >90% of patients, TTP can be fatal if no treatment is received [5]. Refractory TTP is described as a failure to achieve remission in 4-7 days despite initiation of plasma exchange and steroid therapy or gradual worsening of the clinical condition. There is limited literature stating the management of TTP.

## Case presentation

A 62-year-old African American female with a past medical history of hypertension and heavy alcohol abuse was transferred from an outside hospital with worsening mental status and severe thrombocytopenia. Vital signs at the time of admission showed a pulse of 93 beats per minute, blood pressure of 188/114 mm of Hg, respiratory rate of 16 breaths per minute and was saturating 100% on room air. She was awake but oriented only to self. The rest of the physical examination was unremarkable.

Laboratory studies revealed severe thrombocytopenia of 21,000/μL, low hemoglobin of 8.7 gm/dl, and hematocrit of 23.7 % with a normal white cell count. The renal panel revealed an elevated blood urea nitrogen of 54 mg/dl and creatinine of 2.64 mg/dl. The hepatic panel showed alanine aminotransferase of 78 U/l, aspartate aminotransferase of 221 U/l and alkaline phosphatase of 60 U/l. She had elevated indirect bilirubin with negative Coombs test, reticulocyte count was elevated at 4.2%, LDH of 3643 U/l and very low haptoglobin <5.8 mg/dl. Coagulation panel revealed a normal partial thromboplastin time, prothrombin time and reduced fibrinogen levels. Peripheral smear demonstrated numerous schistocytes with polychromasia and severe thrombocytopenia, indicative of microangiopathic hemolysis. Imaging included computed tomography of chest, abdomen, and pelvis which was unremarkable. The activity of vWF protease was severely low at <3% whereas vWF protease inhibitor was high at 2.9 BEU at the time of diagnosis. Further testing included a hepatitis panel which revealed positive hepatitis C virus (HCV) antibody with polymerase chain reaction (PCR) for HCV ribonucleic acid (RNA) showing a viral load of 5,850,000 IU/mL.

**Figure 1:**
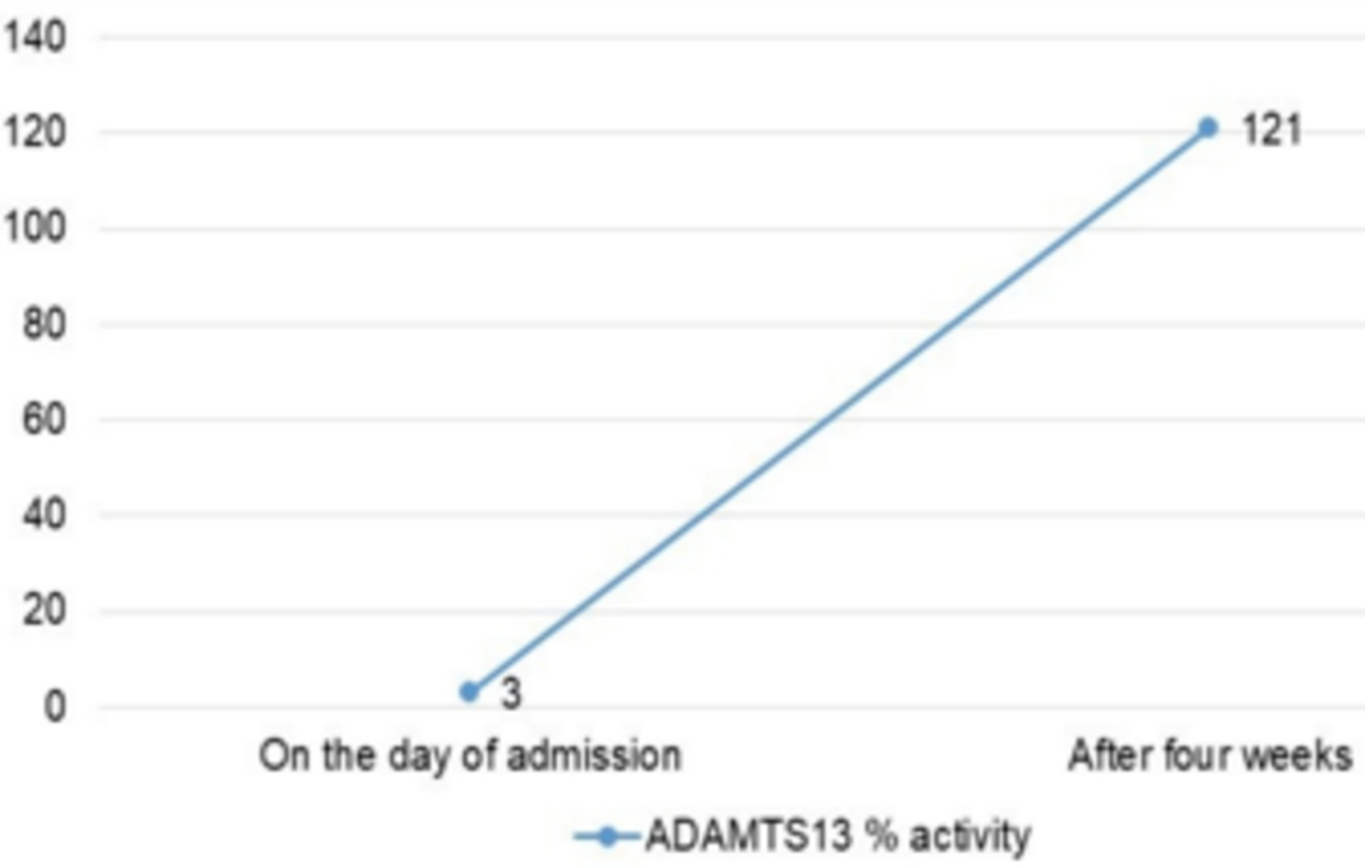
Trend of ADAMTS13 in a duration of one month Rituximab was given around three weeks. ADMAST13 level was drawn after a week of the first dose of rituximab.

**Figure 2:**
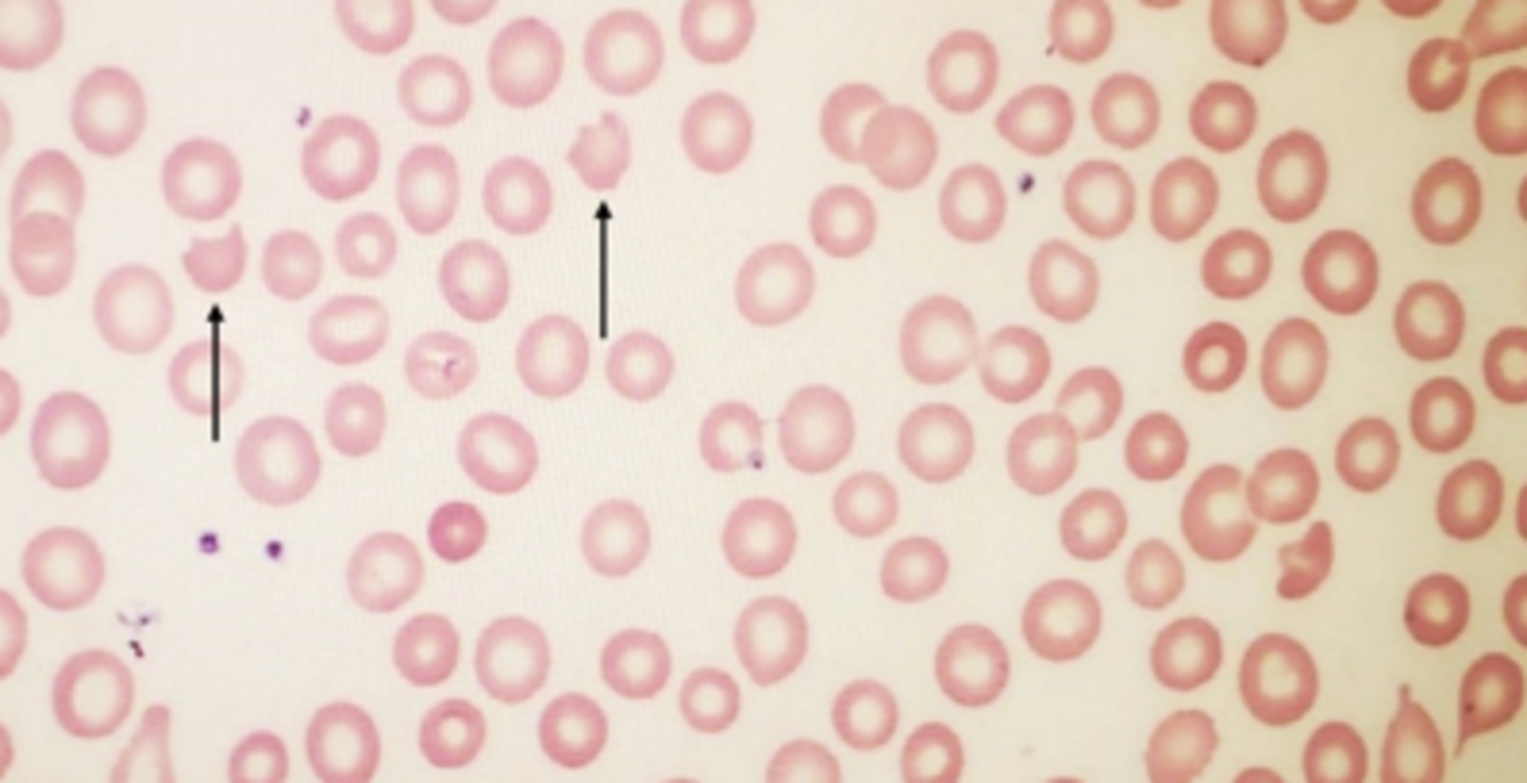
Peripheral smear done after treatment with rituximab revealing fewer schistocytes

**Figure 3:**
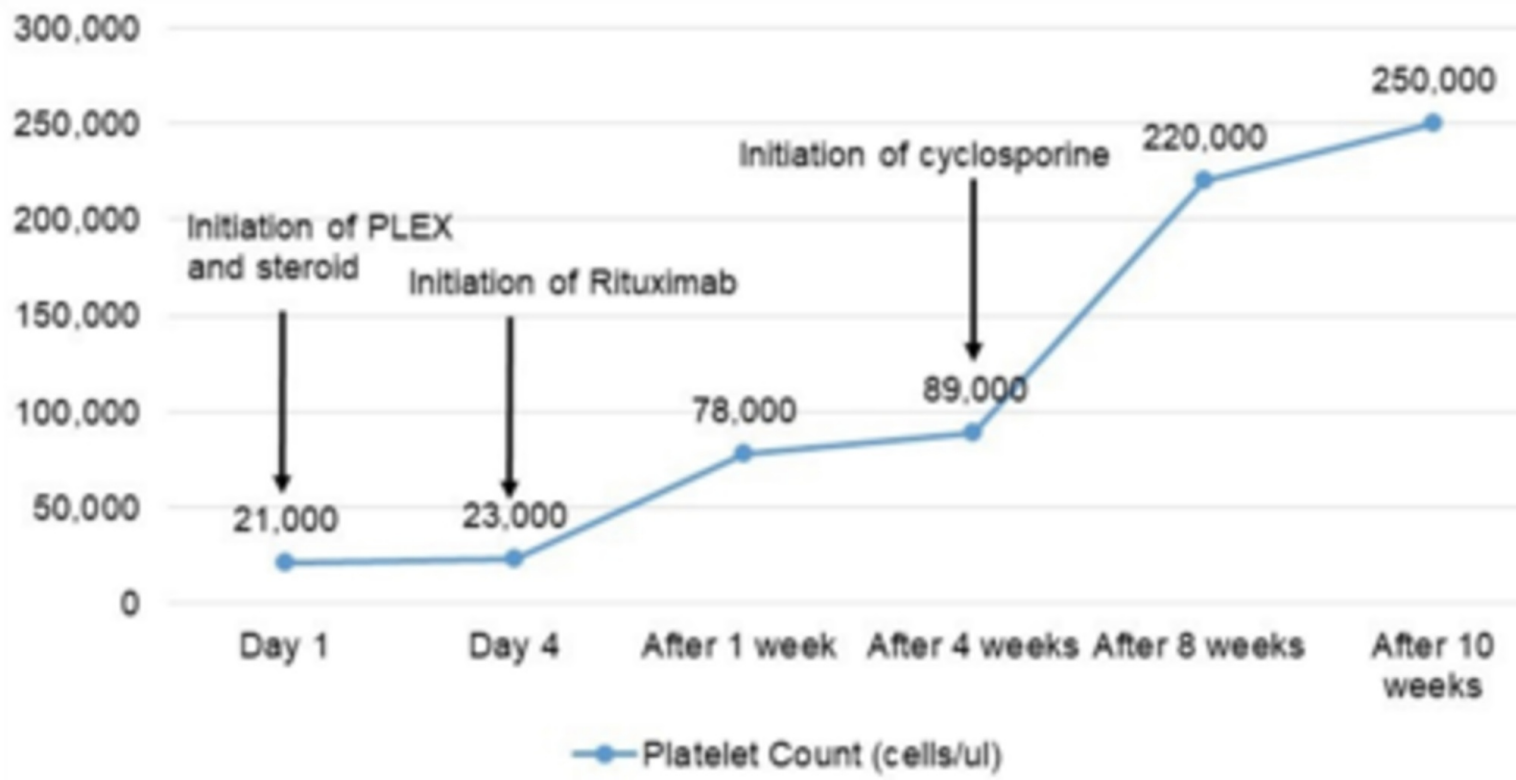
Trends of platelet count over ten weeks Improvement in platelet count was seen after treatment with cyclosporine PLEX: Plasma exchange

At this point, the patient was diagnosed with acquired TTP most likely related to the active hepatitis C infection. Immediately started on PLEX with increased volume of fresh frozen plasma (around 1.5 times the body volume) and prednisone at 1 mg/kg daily. Platelet counts were 23,000/μL on day four of plasma exchange and prednisone. TTP was then considered to be refractory and immunosuppressive therapy with weekly rituximab 375 mg/m2 was started along with plasma exchange. Around the same time, her respiratory status worsened due to pneumonia, which required intubation. She showed quick recovery from the infection and was extubated within 72 hours. She showed clinical improvement by the second week of hospitalization. After a week of rituximab administration, platelet count improved to 78,000/μL along with LDH and reticulocyte count. Peripheral smear revealed the presence of fewer schistocytes. After two weeks of treatment with rituximab, ADAMTS13 activity returned to normal with undetectable vWF inhibitor activity (Figure 1). After completion of four weeks of rituximab, an improvement was seen in the platelet count to 89,000/μL and hematocrit to 28.3 %. Peripheral smear showed few schistocytes. PLEX was then switched from daily to three times a week. She responded to rituximab as evidenced by the appearance of fewer schistocytes in the peripheral smear but not completely as platelet counts continued to remain low (Figure 2). She was then started on the immunomodulatory agent, cyclosporine 50 mg twice daily and two weeks later the platelet count improved to 220,000/μL. Cyclosporine was discontinued after three weeks and the platelet count returned to 250,000/μL (Figure 3).

In total, she received a total of 70 days of PLEX sessions. Follow-up visits revealed that platelet counts, renal function, LDH, reticulocyte count, hemoglobin, and hepatic function panel were all within normal limits. Currently, she is in complete remission and is off treatment. The patient regularly follows up for the management of hepatitis C infection.

## Discussion

Diagnosis of MAHA including TTP is mainly through clinical evaluation, routine laboratory studies and schistocytes on peripheral smear. Our patient was diagnosed with acquired TTP due to her altered mental status along with microangiopathic hemolytic anemia by the presence of schistocytes on peripheral smear, marked thrombocytopenia, high serum LDH and creatinine levels. A decreased ADAMTS13 activity (<3%) and increased ADAMTS13 inhibitor levels (2.9 BEU) further confirmed the diagnosis of TTP, ruling out other causes of thrombocytopenia and MAHA [6].

Infections of bacterial, viral and fungal origin have been reported to precipitate the development of TTP [7]. Viral hepatitis was described as one of the risk factors for TTP [8]. The current serological assays used to identify HCV antibodies cannot definitively distinguish between acute and chronic HCV infection. This is attributed to the fact that viral markers such as anti-HCV immunoglobulin g (IgG) antibody, and HCV RNA may be found in both acute and chronic phase of HCV infection. Clinicians conventionally differentiate acute and chronic HCV infection on the basis of risk factors, clinical presentation, and history of seronegative markers in the past [9-10]. Our patient did not undergo any serological testing in the past and was diagnosed with active hepatitis C infection due to the presence of positive HCV antibody with PCR for HCV RNA showing a viral load of 5,850,000 IU/mL. The serological markers for human immunodeficiency virus (HIV) antibody, total hepatitis B core antibody IgM, hepatitis B surface antigen were all non-reactive.

Current literature identifies the association of chronic hepatitis C and interferon therapy with the development of TTP. lyoda et al. reported a case of TTP that developed during interferon (IFN) therapy in the 16th week, whereas Kitano et al. reported the development of TTP after a month’s therapy with IFN [11-12].

Kamal et al., reported a case of acute hepatitis C that manifested as MAHA resembling TTP and showed resolution of symptoms once the viral infection settled down [13]. We report a case of active hepatitis C associated with serologically proven TTP, which to our knowledge is the first of its kind.

Standard treatment available for TTP is PLEX and corticosteroids. PLEX should be started immediately while awaiting the confirmatory laboratory results. Our patient was started on PLEX and prednisone immediately after peripheral smear showed schistocytes. Equal volumes of fresh frozen plasma are used for PLEX but volumes can be increased to 1.5 times if there is no response or if clinically deteriorating [6].

Our patient was described as having refractory TTP since no response was achieved after treatment with high dose steroids and PLEX after four days of therapy. In the majority of the literature, refractory TTP is described when a patient fails to improve clinically even after standard treatment or failure to normalize platelet count even after 4-7 days of PLEX. The incidence rate of patients with refractory TTP and in need of further management has been reported to be between 10-42% [14]. In such conditions, there is a need to re-evaluate any other causes for thrombocytopenia or anemia. Our patient, however, did not have any obvious cause which could explain the low platelet count.

Rituximab is a monoclonal antibody that targets the B lymphocytes, specifically the CD20 antigen. Literature states that the use of rituximab reduces the duration to achieve response to platelet count, helps to improve ADAMTS13 activity, decreases the extent of PLEX and helps to prevent relapses. After the initiation of rituximab with a dose of 375 mg per week for a total of four weeks, which is the most commonly used dose, her clinical manifestations ameliorated but there was a failure to achieve the desired platelet level [6].

**Figure 4:**
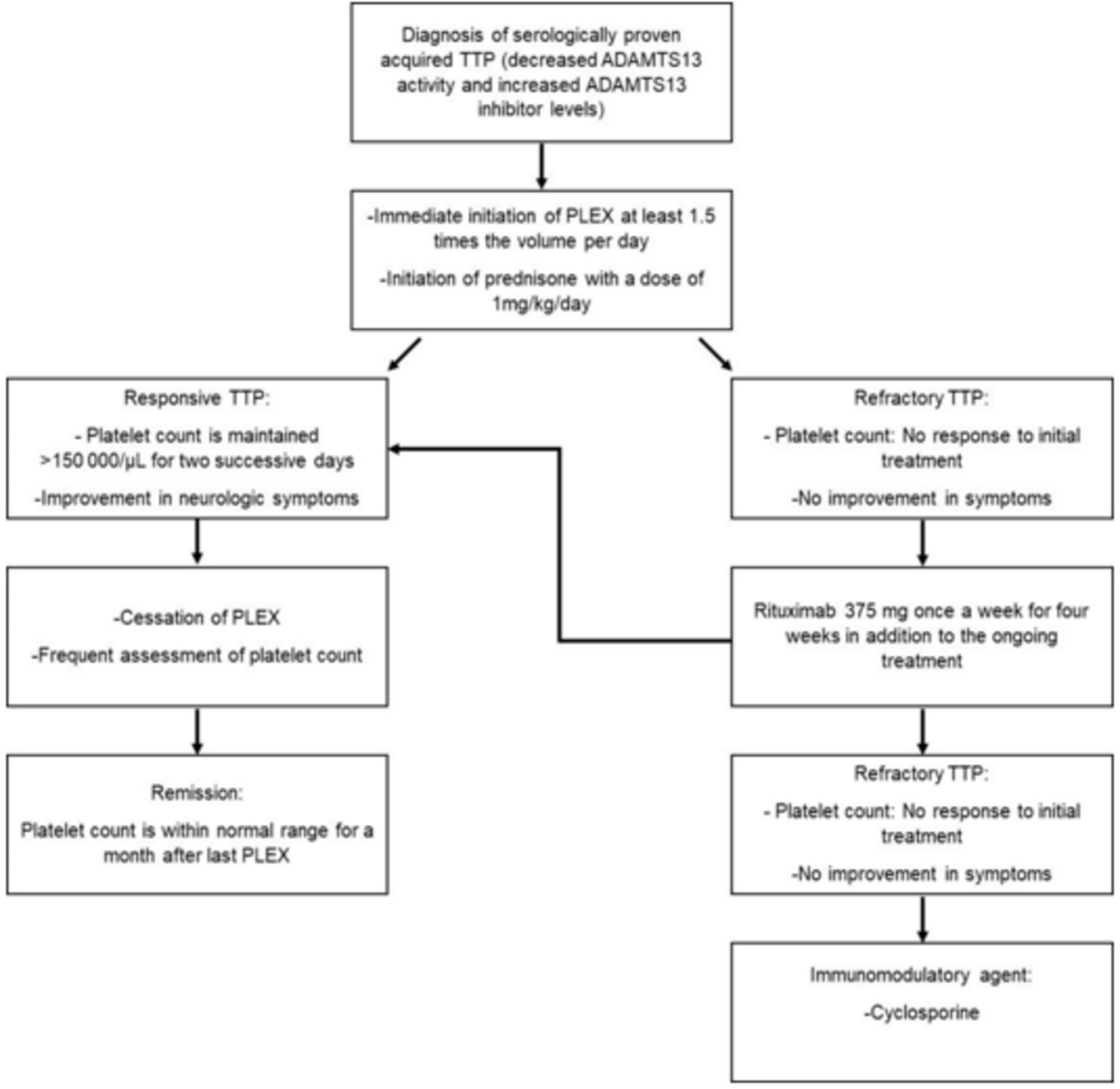
Our approach to the management of refractory TTP Our patient remained refractory to standard therapies like PLEX and steroid administration. Improvement was seen with rituximab and immunosuppressive agents like cyclosporine. TTP: Thrombotic thrombocytopenic purpura; PLEX: Plasma exchange

Treatment with immunomodulatory agents can be considered if refractory TTP persists even after treatment with rituximab [6]. Cyclosporine prevents the activation of T-cell, thus causing inhibition of interleukin-2 (IL-2) receptor expression and eventually decreased the production of IL-2. Cataland et al. revealed that the use of cyclosporine along with PLEX helps in improving ADAMTS13 activity and reducing the activity of ADAMTS13 inhibitor. Out of all the patients involved, 89% achieved remission, proving the efficacy of cyclosporine in refractory cases of TTP as well [15]. Our patient showed significant improvement with combined cyclosporine and PLEX therapy along with steroid administration (Figure 4).

Other treatment options available for refractory TTP include immunosuppressive treatment with cyclophosphamide and vincristine individually as well as in combination. Splenectomy has also been considered as a treatment modality, but data to prove its effectiveness is limited [6].

Recent advances in the field of management for refractory TTP have been reassuring. N-acetylcysteine (NAC) is primarily used in acetaminophen toxicity or in respiratory diseases to reduce the mucinous secretions. vWF is structurally similar to mucin and just like NAC is used to cleave the disulfide bonds in multimers of mucin, it is also postulated that NAC can be used as a replacement to ADAMTS13 and cleave vWF multimers [16]. The usefulness of NAC has been described in the case report by Li et al. involving the treatment of refractory TTP, however, clinical trials are pertinent to prove its clinical efficacy [17]. 

Bortezomib, which is a proteasome inhibitor has reportedly helped to reduce the ADAMTS13 inhibitor levels in refractory TTP. Shortt et al. reported the case of a 53-year-old woman who was diagnosed with TTP unresponsive to prednisone, PLEX, rituximab, cyclophosphamide, and NAC, but showed improvement after treatment with bortezomib [18].

Eculizumab which is a monoclonal antibody has been effective in treatment of complement-mediated thrombotic microangiopathy [19]. However, its role in treatment of acquired TTP has not been established.

The major pitfall with these therapies is that there is limited data to prove their effectiveness and hence more research is needed in this arena. 

## Conclusions

The etiology of TTP is still an enigma in the field of hematology. More extensive studies need to be performed to prove the association between hepatitis C infection and TTP. It is still debatable whether treating hepatitis C infection would prevent further relapses of TTP. In conclusion, timely and appropriate management is required in patients with refractory TTP to prevent fatal consequences.
